# Exfoliation of Transition Metal Dichalcogenides by a High-Power Femtosecond Laser

**DOI:** 10.1038/s41598-018-31374-w

**Published:** 2018-08-28

**Authors:** Sung-Jin An, Yong Hwan Kim, Chanwoo Lee, Dae Young Park, Mun Seok Jeong

**Affiliations:** 1 Department of Energy Science, Sungkyunkwan University, Suwon, 16419 Republic of Korea; 2 Center for Integrated Nanostructure Physics (CINAP), Institute for Basic Science (IBS), Suwon, 16419 Republic of Korea

## Abstract

Thin layer two-dimensional (2-D) transition metal dichalcogenide (TMD) materials have distinctive optoelectronic properties. Therefore, several methods including mechanical exfoliation, chemical vapor deposition, and liquid-phase exfoliation have been attempted to obtain uniform TMDs. However, such methods do not easily produce high-quality few-layer TMDs with high speed. Here, we report the successful fabrication of few-layer TMD materials by femtosecond laser irradiation. It shows that TMD samples can be exfoliated from bulk to ~3 layers. This method is much faster and simpler than other exfoliation methods. The size and number of the layers were confirmed by atomic force microscopy, scanning electron microscopy, Raman spectroscopy, and photoluminescence experiments. It is expected to be used for the mass production of thin 2-D TMD materials.

## Introduction

Atomically thin-layered materials such as graphene and transition metal dichalcogenides (TMDs) are of interest because of their resilient electronic, optical, and catalytic properties^1,2^. In particular, thin-layered two-dimensional (2-D) materials have many advantages such as high electron mobility, high thermal conductivity, and a direct bandgap at the monolayer^3–6^. Accordingly, many researchers have attempted to fabricate atomically thin 2-D materials including monolayers through various methods such as mechanical exfoliation and chemical vapor deposition (CVD). Mechanical exfoliation with a scotch tape produces high-quality samples, though it takes too long time to find few-layer samples using an optical microscope because of its low yield^7–9^. On the other hand, CVD produces samples that can be controlled in terms of the size and number of layers; however, such samples have lower optical and electronic properties than the samples fabricated by mechanical exfoliation method^10–12^. Moreover, these methods require an additional dry or wet transfer process for the device application^7,13^. Therefore, a drop-casting method after exfoliation in solution was studied to avoid the transfer process^14,15^. The exfoliation process in liquid leads to faster preparation method. However, the samples made by exfoliating in solution are extremely small, and it is difficult to control the layer number and size of the flakes^16,17^. Recently, it has been reported that the exfoliation of graphene in liquids by a nanosecond (NS)-pulse Nd:YAG laser is more effective for obtaining a large-sized and high-quality sample than the other liquid exfoliation methods^18^. However, exfoliation by laser has not yet been applied to the TMD materials. Because, it is difficult to optimize the conditions of the laser depending on the materials and focus the laser on the materials surface. Also, the sample is not exfoliated or easily damaged in the sample depending on the laser power. In the case of TMD materials, the pulse laser has been used to fabricate electro catalysts, quantum dots, and MoO_x_ materials^19–21^. However, there are several reports that NS laser irradiation can generate lots of defects because of heat dissipation. When the laser focused by the convex lens is irradiated on the surface, the strong thermal energy is generated at the contact point. The irradiated area is deformed due to excessive heat energy, and the position will become defective in the laser exfoliation process. In this experiment, we have studied the exfoliation of the bulk 2-D material to fabricate the thin layers using femtosecond (FS) laser rather than NS laser, because FS laser which has short pulse duration time can minimize the defects caused by heat that can be occurred in laser irradiation process^22,23^. Using this method, the bulk TMD materials were successfully exfoliated with the several-layered samples. Atomic force microscopy (AFM), scanning electron microscopy (SEM), confocal Raman spectroscopy, and photoluminescence (PL) have been used to identify the size and number of layers of FS laser exfoliated sample.

## Results and Discussion

**Figure 1:**
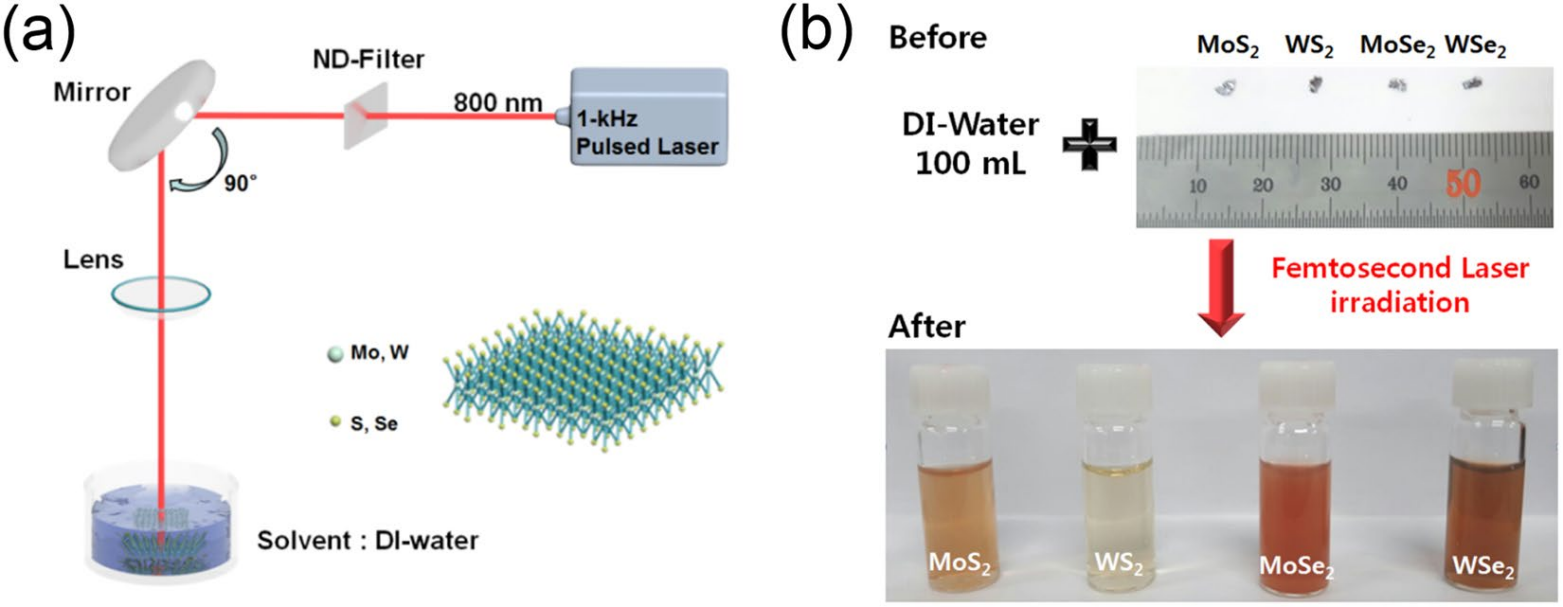
(**a**) Experimental setup for the laser exfoliation of bulk TMD materials. (**b**) Schematic representation of the preparation and exfoliation of a 2-D nanosheet solution from layered bulk materials. The photograph titled “After” reveals dispersed solutions with the different 2-D nanosheets.

**Figure 2:**
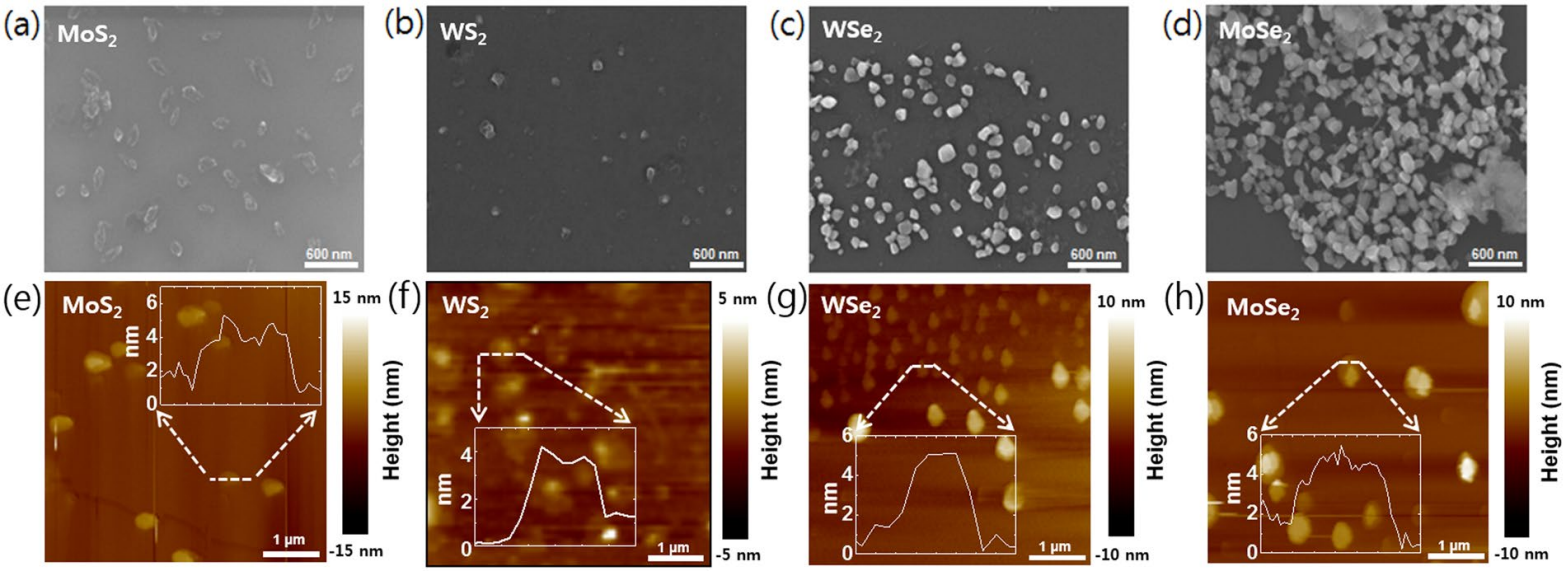
Laser exfoliated few-layer TMD nanosheets on SiO_2_/Si substrate. The figures (**a**–**d**) and (**e**–**h**) represent the SEM and AFM images of few-layer MoS_2_, WS_2_, MoSe_2_, and WSe_2_, respectively. The insets in (**e**–**h**) represent the height profiles of the TMD flakes.

**Figure 3:**
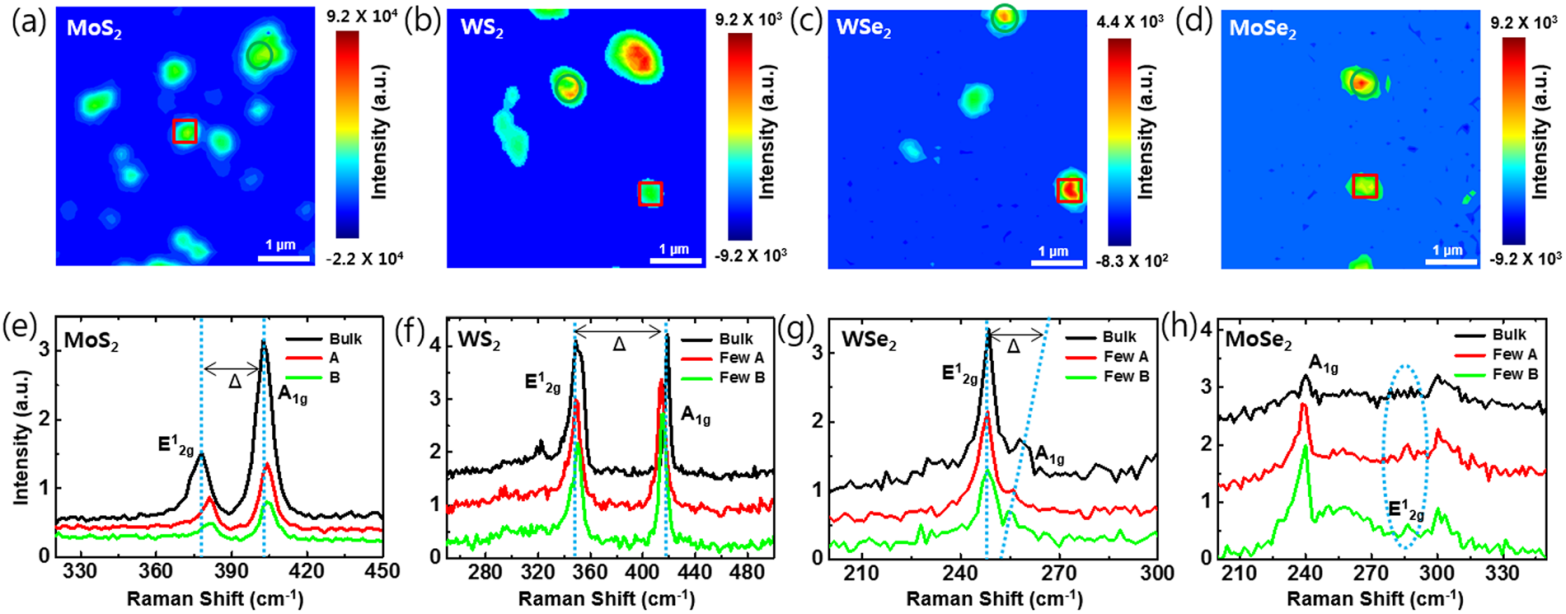
Raman scattering data of the TMD materials. The figures (**a**–**d**) represent the Raman scattering mapping of the TMD flakes after laser exfoliation. These mapping data indicate the Raman images of E^1^_2g_ and A_1g_ peaks area intensity. The figures (**e**–**h**) represent the Raman spectra of thin-layer TMD nanosheets and bulk materials.

The liquid-phase exfoliation method has attracted interest from many research groups because it is simpler and faster than the other exfoliation methods. Moreover, a liquid-phase exfoliated sample can be placed in the desired position. Likewise, the laser exfoliation method in a liquid solution offers similar advantages with a higher productivity. In the laser exfoliation process, however, samples could be damaged or deformed by excessive laser irradiation. Therefore, we performed laser exfoliation under optimized laser power (1.2 W) at a constant incident angle (90°) in DI water (100 mL) to avoid significant damage (Figs S1, 2). In this case, we observed that the TMD materials were found to be exfoliated with a constant size and number of layers. Also, irradiation time dependent absorption measurement was performed to optimize the condition of exfoliation process (Fig. S4). In our experiment, we used single crystals of molybdenum disulfide (MoS_2_), molybdenum diselenide (MoSe_2_), tungsten disulfide (WS_2_), and tungsten diselenide (WSe_2_) for laser exfoliation. Our experimental setup is shown in Fig. 1a. An amplified Ti:sapphire laser of 80-fs pulse duration with 1-kHz repetition rate and 800-nm center wavelength was focused on the surface of bulk TMD materials. The beam spot size was around 5 mm in diameter, and ethanol, deionized water, and N-methyl-pyrrolidone (NMP) were used as a solvent to suspend the exfoliated sheets. Figure 1b shows the procedure of sample preparation and the exfoliated samples in solution after laser irradiation. Approximately 0.01 g of the sample was placed in 100 mL of the solvent before being directly irradiated by the laser. The color of the solution is drastically changed upon irradiation, directly indicating that the sample was physically affected by laser irradiation. Exfoliated samples were placed on the SiO_2_/Si substrate via drop-casting in order to identify the thickness of the sample. The morphology and characteristics of the dispersed samples obtained by exfoliation were carefully studied using SEM and AFM. Figure 2a–d shows the SEM images after laser exfoliation. These images show clearly that the sample was distributed as nanosheets on the silicon substrate. Furthermore, Fig. 2a shows an individual exfoliated MoS_2_ sheet with a lateral size of ~200 nm. Similarly, Fig. 2b–d shows the typical SEM images of WS_2_ (~200 nm), MoSe_2_ (~100 nm), and WSe_2_ (~200 nm) sheets, respectively. In addition, we randomly selected more than 50 nanosheets of various TMD materials (MoS_2_, WS_2_, MoSe_2_, and WSe_2_) and analyzed them with AFM to obtain their average thicknesses. As shown in the AFM height profile in Fig. 2e–h, micron-sized areas with heights in the nanometer range are clearly identified. As a result, our laser irradiated TMD material appears clearly exfoliated^24^. It is well known that the Raman spectroscopy is a powerful analytical tool for determining the thickness and stacking of 2-D TMD layered materials^25^. Figure 3 shows the Raman scattering image and spectra of the exfoliated samples and bulk TMD material. The specific areas shown in the Raman mapping image are displayed the same color in the Fig. 3e–h. Also, Raman spectrum of bulk sample was indicated by a black line. The spectrum named with Few-A and B were extracted from the marked region with circle and square in the Fig. 3e–h. We observed both the E^1^_2g_ and A_1g_ modes in the few-layer TMD materials upon laser irradiation. E^1^_2g_ and A_1g_ are the only intense modes that correspond to the in-plane (interlayer) and out-of-plane (interlayer) vibrations, respectively^26^. In MX_2_ materials (M = Mo, W and X = Se, S), the spacing between the two modes decreases as material thickness decreases, which is an excellent indicator of the number of layers^27,28^. Figure 3e–g spectra show the distance between E^1^_2g_ and A_1g_ modes of bulk and exfoliated samples^25,29,30^. For WSe_2_, two dominant peaks are observed around 250 cm^–1^ in various samples from monolayer to bulk (see Fig. 3g). In the case of bulk MoSe_2_, a weak vibrational E^1^_2g_ mode was observed at 286 cm^−1^. The intensity of this mode was gradually increased after laser irradiation. This indicates that bulk MoSe_2_ was successfully exfoliated to few-layer^25^.

**Figure 4:**
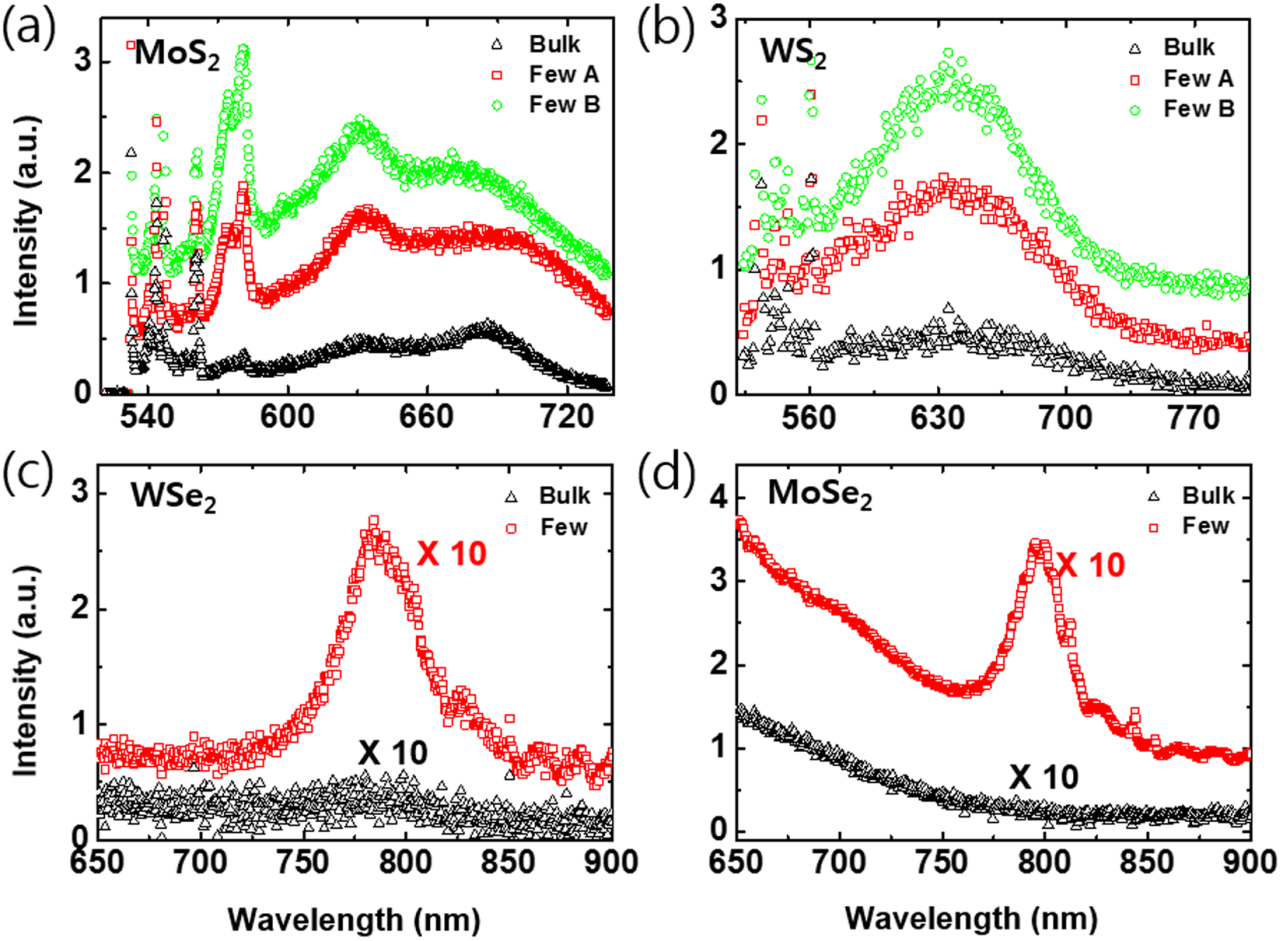
PL spectra of TMDs before and after laser exfoliation process; (**a**) MoS_2_, (**b**) WS_2_, (**c**) WSe_2_, (**d**) MoSe_2_. Few A (red square) and B (green circle) in PL spectra of (**a**,**b**) were exported from Figure S3. PL spectra of (**c**) WSe_2_ and (**d**) MoSe_2_ were acquired from point measurement and bulk spectra (black triangle) of (**a**–**d**) were obtained from bulk flakes before laser irradiation process.

Figure 4a–d shows the photoluminescence (PL) spectra after the laser exfoliation of MoS_2_, WS_2_, MoSe_2_ and WSe_2_, respectively. Figure 4a,b are from the area marked with circle and square of PL mapping image of MoS_2_ and WS_2_ in Figure S3. In the case of MoSe_2_ and WSe_2_, we could not achieve PL mapping images because of their low Pl efficiency. Generally, thin-layer TMDs become a direct band gap semiconductor and the photoluminescence intensity drastically increases with a decreasing number of layers^25,31–33^. The MX_2_ materials which are consisted of one transition metal (M) sandwiched by two chalcogen atom (X) are formed into layered structure with van der Waals interaction. In case of bulk MX_2_, the electronic states involved in the indirect band-gap transition originate from linear combinations of molybdenum, tungsten (Mo, W)-orbitals and sulfur, selenium (S, Se)-orbitals. These electronic states exhibit strong interlayer coupling and their energy is quite dependent on the number of layers. Therefore, PL measurement is a simple way to confirm the change in the thickness of TMDs. Figure 4a–d show that the TMDs materials has a higher PL signal due to the change in valence and conduction bands after laser irradiation^32,34^. It was plainly see the change in the conduction and valence band in TMD materials after laser irradiation. These results are clear evidence for the successful exfoliation of the sample. Through PL and Raman data at the same area, we confirmed that the tendencies of both spectra are almost identical. These results show that the TMD materials were changed to few-layer thickness by laser irradiation for 1 h. At last, we have obtained the samples with 3~4 layers by FS laser exfoliation (DI-water for 1 hour with a laser power intensity of 1.2 W) and it is confirmed through AFM, Raman and PL experiments.

**Figure 5:**
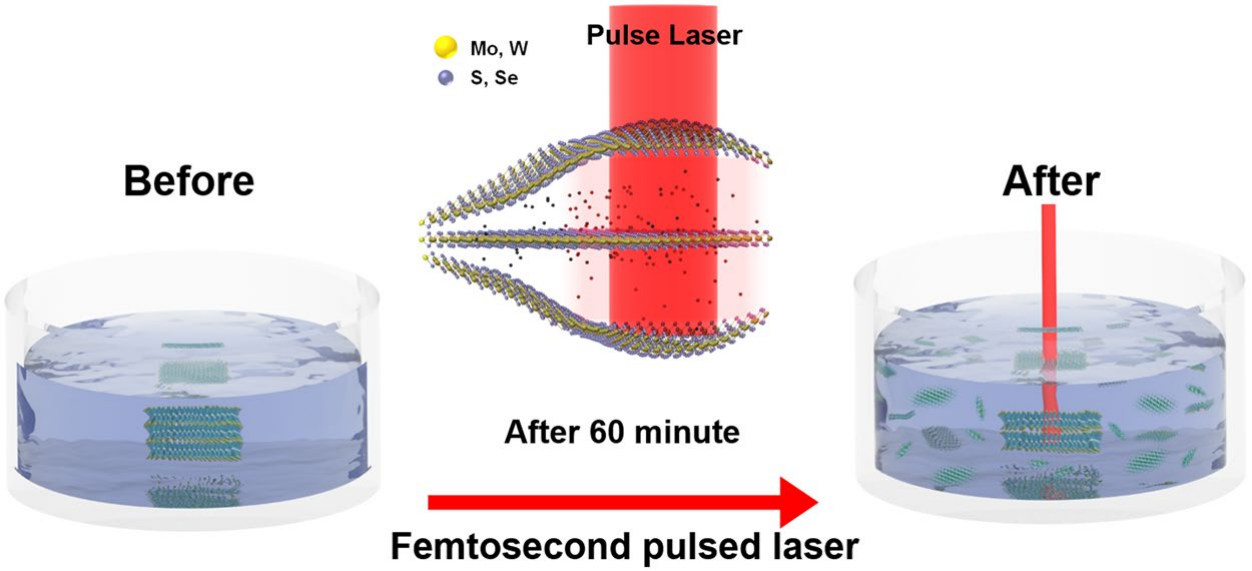
The laser exfoliation mechanism of the TMD materials. Schematic of the ‘Before’ and ‘After’ laser exfoliation process. (Laser power_1.2 W, 60 minute_ laser irradiation time and Di-water based solvent).

Figure 5 shows the conceptual model of the mechanism of this experiment. Layered TMD formed MX_2_ crystallized in a strongly covalent bond X–M–X sandwich structure. Consecutive X–M–X layers are weakly bound by van der Waals interactions. The TMD materials appear in different formations that are distinguished by their stacking orders. For this reason, previous exfoliation methods such as sonication, centrifugation, and cavitation, should have specific experimental conditions for the exfoliation of various TMD materials. However, in the laser exfoliation method, it is possible to break the van der Waals interactions between layers of different TMD materials using the same experimental condition. In other words, several different conditions are not needed for exfoliating different TMDs. This laser exfoliation method makes it possible to manufacture a specific number of layers very easily in a relatively short time (~1 h) than the conventional exfoliation method. In addition, because the liquid-phase separation process using a laser can produce a large amount of sample, it is expected that several layers of the TMD solution can be obtained more easily. However, when increasing the laser power or irradiation time to obtain a mono or bi-layer, the layers were split into small size or different form (Quantum-dots and MoO_X_ materials) caused by strong heat energy of laser. Therefore, further efforts to find optimized condition for fabricating more thin layers should be required to enhance the optical properties and expand the applications of exfoliated TMD.

## Conclusions

We exfoliated TMDs (MoSe_2_, MoS_2_, WS_2_, and WSe_2_) by femtosecond laser irradiation for 1 hour in a liquid solution. This process is much faster than the conventional liquid-phase exfoliation methods that combine sonication and centrifugation. The exfoliated TMD materials consist of few-layer in an arbitrary shape with a feature size of 100 to 200 nm. Through an optical analysis of 2-D photoluminescence and Raman mapping, we confirmed that the laser irradiated TMDs have homogeneous few-layer. This method has advantage for the mass production of few-layered TMD materials. Thus, it can be used for large area optoelectronic devices such as photodetector and photovoltaic devices^35,36^.

## Methods

### Laser exfoliation process

The exfoliated TMD flakes were prepared using a homemade laser irradiation system as shown in Fig. 1a. In this system, the laser (Coherent Libra Integrated Ti:S Amplifier) to be focused is passed to gold mirror and focusing lens (Thorlabs, Inc.). The focal length of the lens used for focusing was 150 mm, the diameter of the laser beam before passing through the lens was about 12 mm. Bulk TMD materials (2-D Semiconductor) before exfoliation was approximately 0.01 g, placed in Deionized-water and laser treated for 1 hour. We used special methods to prevent aggregation of the material during drying of the aqueous solution after laser exfoliation. The solution containing few-layer TMD materials obtained from the experiment was processed at 5000 rpm five times (each round of duration 10 min) to create a new solution with a higher concentration of TMD layers. The solution obtained was drop cast on a silicon substrate by blowing N_2_ gas at 3000 rpm using a spin coater. An atomic force microscopy (Nano IR + AFM SYSTEM) setup was used in contact mode, the scan rate was 0.2 Hz, and the step resolution 50 nm. All the measurements were performed at room temperature. Confocal Raman spectroscopy (XperRam 200) and a confocal micro-photoluminescence setup were used to excite the sample with a continuous-wave laser (λ = 532 nm) that was focused using a 40× objective lens (NA = 0.75). The Raman scattering results were collected with the same objective that was dispersed with f = 200 mm spectrometer.

## Electronic supplementary material

Supplementary information
