# Supplementary material for: Exfoliation of Transition Metal Dichalcogenides by a High-Power Femtosecond Laser
Source: Sci Rep. 2018 Aug 28;8:12957. doi: 10.1038/s41598-018-31374-w (PMC6113326; doi:10.1038/s41598-018-31374-w)
Supplement: Supplementary file 1 — Supplementary information [file 41598_2018_31374_MOESM1_ESM.docx]

**Supplementary Information**

**Exfoliation of Transition Metal Dichalcogenides by a High-Power Femtosecond Laser**

Sung-Jin An^1,2,†^, Yong Hwan Kim^1,†^ Chanwoo Lee^1,2^, Dae Young Park^1,2^ and Mun Seok Jeong^1,2,*^

^1^Deparment of Energy Science, Sungkyunkwan University, Suwon 16419, Republic of Korea;

^2^Center for Integrated Nanostructure Physics (CINAP), Institute for Basic Science (IBS),

Sungkyunkwan University, Suwon 16419, Republic of Korea

^†^ These authors contributed equally to this work.

*Correspondence and requests for materials should be addressed to M.S.J. (email: mjeong@skku.edu)

**Supplementary Figures**

1. **The power dependence of the laser exfoliation method**
2. **Raman scattering of exfoliated MoS_2_ in different aqueous solutions**
3. **Photoluminescence mapping of exfoliated MoS_2_ and WS_2_**
4. **Irradiation time-dependent absorption of exfoliated MoS_2_ by laser exfoliation process**

**Supplementary References**

**Supplementary Figures**

**S1. The power dependence of the laser exfoliation method**

We confirmed the dependence of the laser power on exfoliated MoS_2_ in DI water from the Raman measurements. The material did not exfoliate when irradiated with 0.5 W of a weak power laser. However, for a laser output greater than 2 W, the sample was damaged and could not detect the Raman signals. Consequently, the power of the laser was determined as the most important factor in the exfoliation process.


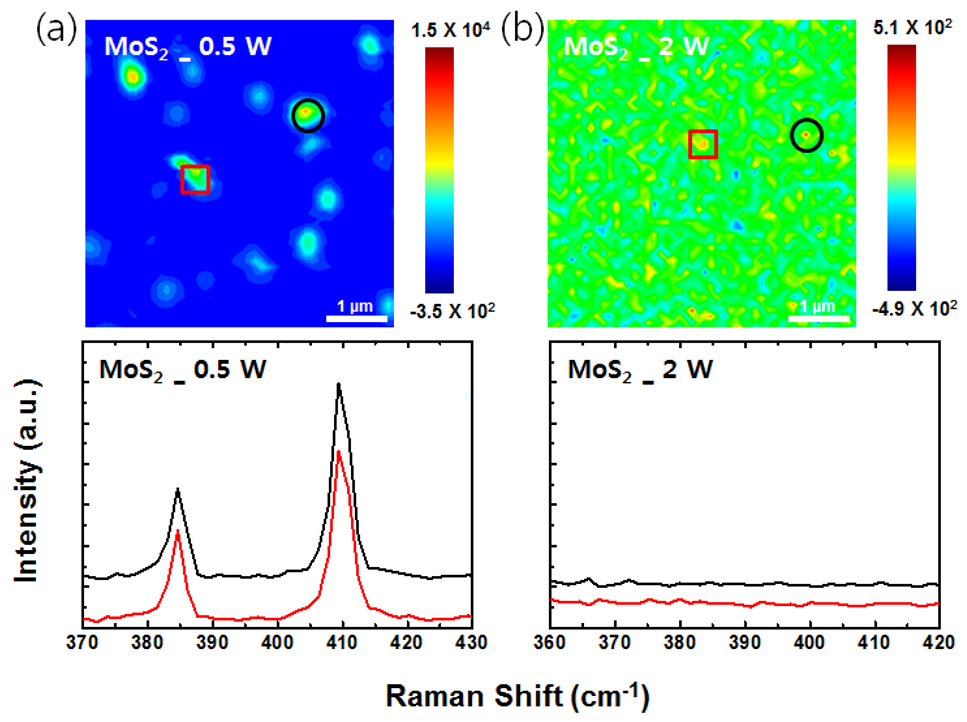


**Figure S1.** Laser exfoliation for different laser powers: (a) 0.5 W and (b) 2 W.

**S2. Raman scattering of exfoliated MoS_2_ in different aqueous solvents**

We examined different solvents (NMP and ethanol) for the laser exfoliation method (1 hour irradiation) and confirmed the number of layers of MoS_2_ with Raman scattering^[1]^. The experimental conditions are same with laser irradiation in DI water. From the Raman scattering data, positions of A_1g_ and E^1^_2g_ of the exfoliated flake are not changed a lot compared to bulk material. It indicates that DI water is more appropriate solvent for achieving few-layer exfoliated flakes in the same time.


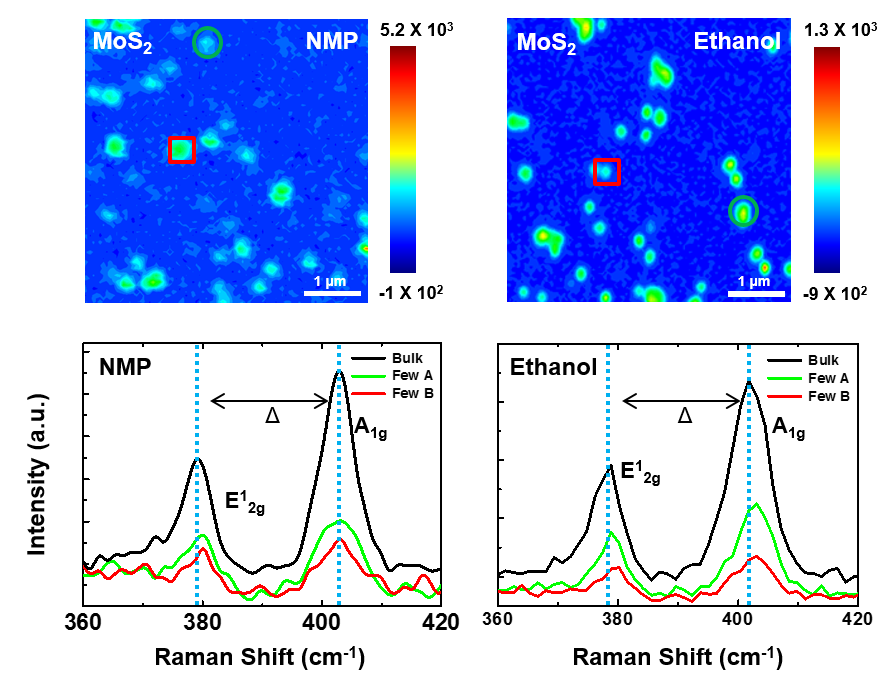


**Figure S2.** The results of laser exfoliation based on N-methyl-pyrrolidone (NMP) and ethanol solutions.

**S3. Photoluminescence mapping of exfoliated MoS_2_ and WS_2_**

We confirmed the photoluminescence mapping of the MoS_2_ and WS_2_ flakes prepared by laser irradiation. Measured area are same with the Raman mapped image in Figures 3a and b. The PL spectra in Figure 4 are extracted from the area marked with circle and square. In the case of the other TMDs (MoSe_2_ and WSe_2_), mapping was impossible due to low photoluminescence intensity.


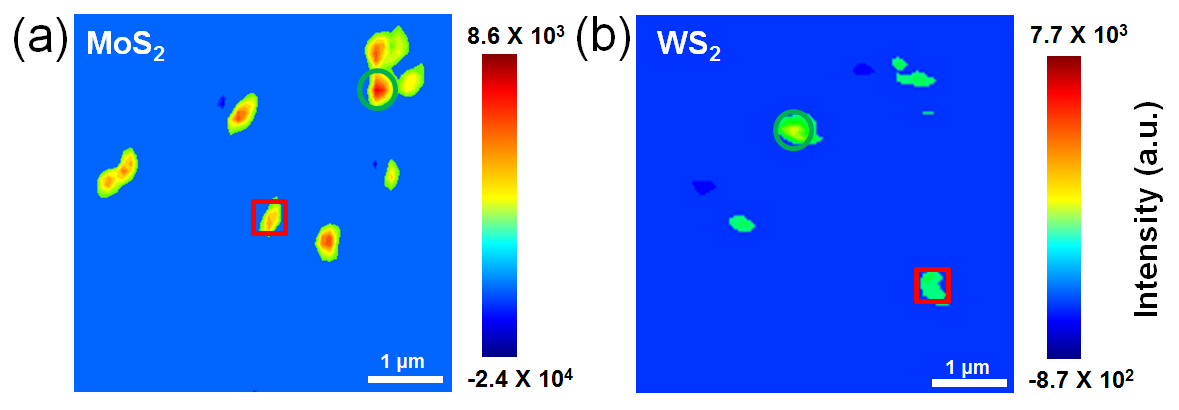


**Figure S3.** Photoluminescence mapping of the TMD (MoS_2_ and WS_2_) flakes after laser exfoliation.

**S4. Irradiation time-dependent absorption of exfoliated MoS_2_ by laser exfoliation process**


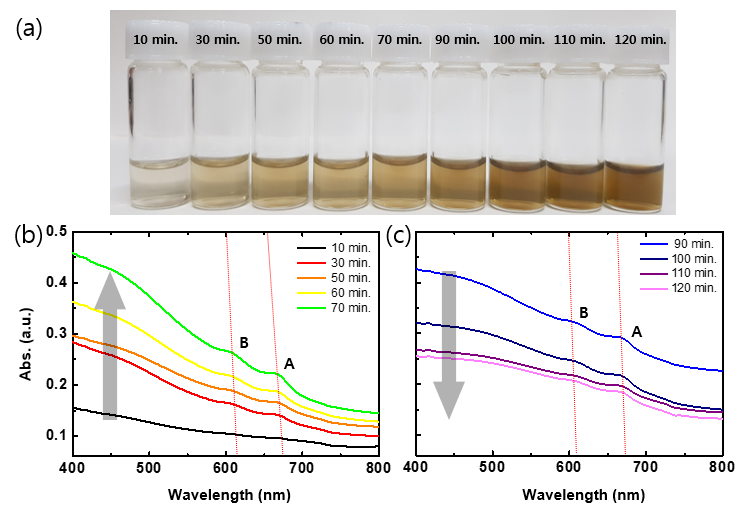
We performed the irradiation time dependent absorption measurement of the MoS_2_ solution (from 10 to 120 minutes). The samples in figure (a) were made under the same condition in Figure 1b (1.2 W; laser power and DI-water based solvent). Figure (b) and (c) have two noticeable broad peaks at wavelengths of ~ 610 nm and ~ 670 nm. These correspond to the absorption peaks generated by electronic transitions at the K point of the Brillouin zone associated with the formation of B and A excitons. As shown in the figure, B and A exciton are shifted to higher energy which indicates the thinning of MoS_2_ layer. When the irradiation time of the laser is more than 80 minutes, the absorption intensity decreases. We assume that the material is damaged by the laser irradiation for a long time^2,3^_._ Thus, we adopted 60 minutes irradiation time for the high productivity of exfoliation with low irradiation damage.

**Figure S4.** Irradiation time-dependent absorption spectra of the exfoliated MoS_2_ solutions; (a) photograph of exfoliated MoS_2_ solution. The figures (b) and (c) indicate the absorption spectra of samples in figure (a), respectively. The labels A and B indicate the name of the exciton peaks.

**Supplementary References**

1 J.-H. Fan, et al. Raman scattering in bulk 2H-MX_2_ (M = Mo, W; X = S, Se) and monolayer MoS_2_. *Journal of Applied Physics* **115**, 053527 (2014).

2 Mak K F, et al. Atomically thin MoS_2_: a new direct-gap semiconductor. *Phys. Rev. Lett.* **105,** 136805 (2010).

3 Splendiani A, et al. Emerging photoluminescence in monolayer. MoS_2_ *Nano Lett.* **10,** 1271–5 (2010).
